# Atrial slow conduction develops and dynamically expands during premature stimulation in an animal model of persistent atrial fibrillation

**DOI:** 10.1371/journal.pone.0258285

**Published:** 2021-10-07

**Authors:** Matthias Lange, Annie M. Hirahara, Ravi Ranjan, Gregory J. Stoddard, Derek J. Dosdall

**Affiliations:** 1 Nora Eccles Harrison Cardiovasular Research and Training Institute, University of Utah, Salt Lake City, Utah, United States of America; 2 Biomedical Engineering, University of Utah, Salt Lake City, Utah, United States of America; 3 Division of Cardiovascular Medicine, Department of Internal Medicine, University of Utah School of Medicine, Salt Lake City, Utah, United States of America; 4 Division of Epidemiology, Department of Internal Medicine, University of Utah School of Medicine, Salt Lake City, Utah, United States of America; 5 Division of Cardiothoracic Surgery, Department of Surgery, University of Utah School of Medicine, Salt Lake City, Utah, United States of America; Kurume University School of Medicine, JAPAN

## Abstract

Slow conduction areas and conduction block in the atria are considered pro-arrhythmic conditions. Studies examining the size and distribution of slow conduction regions in the context of persistent atrial fibrillation (AF) may help to develop improved therapeutic strategies for patients with AF. In this work, we studied the differences of size and number in slow conduction areas between control and persistent AF goats and the influence of propagation direction on the development of these pathological conduction areas. Epicardial atrial electrical activations from the left atrial roof were optically mapped with physiological pacing cycle lengths and for the shortest captured cycle lengths. The recordings were converted to local activation times and conduction velocity measures. Regions with slow conduction velocity (less than $0.2\frac{m}{s}$) were identified. The size of the connected regions and the number of non-connected regions were counted for propagation from different orthogonal directions. We found that regions of slow conduction significantly increases in our 15 persistent AF goat recordings in response to premature stimulation (24.4±4.3% increase to 36.6±4.4%, *p* < 0.001). This increase is driven by an increase of size from (3.70±0.89[*mm*^2^] to 6.36±0.91[*mm*^2^], *p* = 0.014) for already existing regions and not by generation of new slow conduction regions (11.6±1.8 vs. 13±1.9, *p* = 0.242). In 12 control goat recordings, no increase from baseline pacing to premature pacing was found. Similarly, size of the slow conduction areas and the count did not change significantly in control animals.

## Introduction

Atrial fibrillation (AF) is the most frequently diagnosed chronic arrhythmia [1] and is often a recurring condition, while its reversal is slow and limited [2–7]. Despite significant advances in management strategies of rhythm and rate control, the long-term efficacy of treatments such as drug therapy and catheter ablation are inconsistent. Identification of patients who would benefit most from these specific therapies would be a significant advance in care for AF patients.

Collagenous tissue has been linked to conduction disturbances and it is well understood that fibrosis is a major contributor to the maintenance of AF [8]. Investigators have recreated the 3-dimensional structure of collagenous septae in ventricular tissue [9–11] and demonstrated that these collagenous structures play a significant role in conduction and act as secondary sources of excitation during defibrillation [12–14]. It has been hypothesized that different types of atrial fibrosis lead to different conduction abnormalities: diffuse fibrosis may lead to overall conduction slowing, patchy fibrosis may lead to unidirectional block, and stringy interstitial fibrosis may lead to slowed or blocked transverse conduction [15].

Similarly, Angel et al. hypothesized that specific fibrosis structures enable AF [16]. To study this, they measured the conduction velocity in atrial tissue of control goats and rapidly-paced goats with 6 months of AF. In an open chest study, they placed an array of 256 electrodes on the left atrial appendage and delivered S1-S2 pacing from the center of the array. The anisotropy between control and AF goats was similar in S1 beats (1.55±0.42 vs. 1.57±0.33) but changed for S2 beats (1.72±0.55 vs. 1.47±0.25). After the electrophysiological study 2-dimensional histology was conducted to evaluate the local fibrosis pattern, showing a higher interstitial or disruptive fibrosis density for AF goats.

Conduction velocity is not homogeneously affected by the shorting of the pacing interval. Spach et. reported an exponential decrease in conduction velocity in transversal direction for coupling intervals between 100ms and 300ms, while the longitudinal direction was not significantly changed [17]. This findings were recently confirmed in a human case report [18] and is in agreement with Angel et al. [16]. A similar directionally dependent response can be seen in the development of conduction block; specifically, longitudinal conduction is more likely to be disrupted and block compared to transverse conduction [19].

Aside from the direction of propagation, short coupling intervals between activations promotes slow condition (SC), fractionation, and the development of conduction block [8, 18]. The developed conduction block can be unidirectional, combining the cycle length dependency and the directional dependency. The differences in conduction between directions makes directionality of propagation an important factor when investigating SC or blockage.

It has been shown that fibrotic tissue is associated with SC, which can also be rate or directionally dependent. However, quantification of how much tissue is affected by the SC remains unclear. Therefore, we set out to investigate the size and quantity of SC areas. We will answer the question of how the size of affected areas change in response to different pacing protocols and locations and how these changes differ between healthy control goats and goats with persistent AF.

## Materials and methods

Animal experiments were approved by the Institutional Animal Care and Use Committee of the University of Utah and followed the Guide for the Care and Use of Laboratory Animals [20].

### Animal model

In total, 55 pace maps where obtained from 7 goats (3 female); 4 goats (2 female) formed the persistent AF model. For the generation of the persistent AF model goats were first anesthetized with propofol (5-10mg/Kg iv) and anesthesia was maintained by ventilation with a mix of oxygen (2 l/min) and isoflurane (2-4%). Following a previous established procedure, a catheter was then advanced from the right jugular vein to the right atrium where a pacing lead was secured [21]. A pacemaker (InterStim™, Medtronic, Minneapolis, MN) was connected to a lead in the right atria. Pacing was initiated one week following pacemaker implant and configured for 50Hz pacing with one second on followed by one second off. The pacing was continued until AF was sustained for more than one minute, at which point pacing was reduced to one second on and 30 seconds off. From this point onward the animals were constantly in AF for a total of at least six months, as ensured by weekly ECG readings. For the study, a sternotomy was performed under general anesthesia and 5000 units of Heparin was injected. The animal was euthanizing by exsanguination. First, the aorta was clamped and than the heart was quickly excised and immediately submerged in ice-cold Tyrod solution (NaCl 5.2mM, NaH_2_PO_4_-H_2_O 48*μ*M, MgCl_2_-6H_2_O 40*μ*m, CaCl_2_-2H_2_O 72*μ*M, NaHCO_3_ 0.96mM, Dextrose 11.20mM, Bovine Serum Albumin 0.60*μ*M).

### Optical mapping

**Fig 1:**
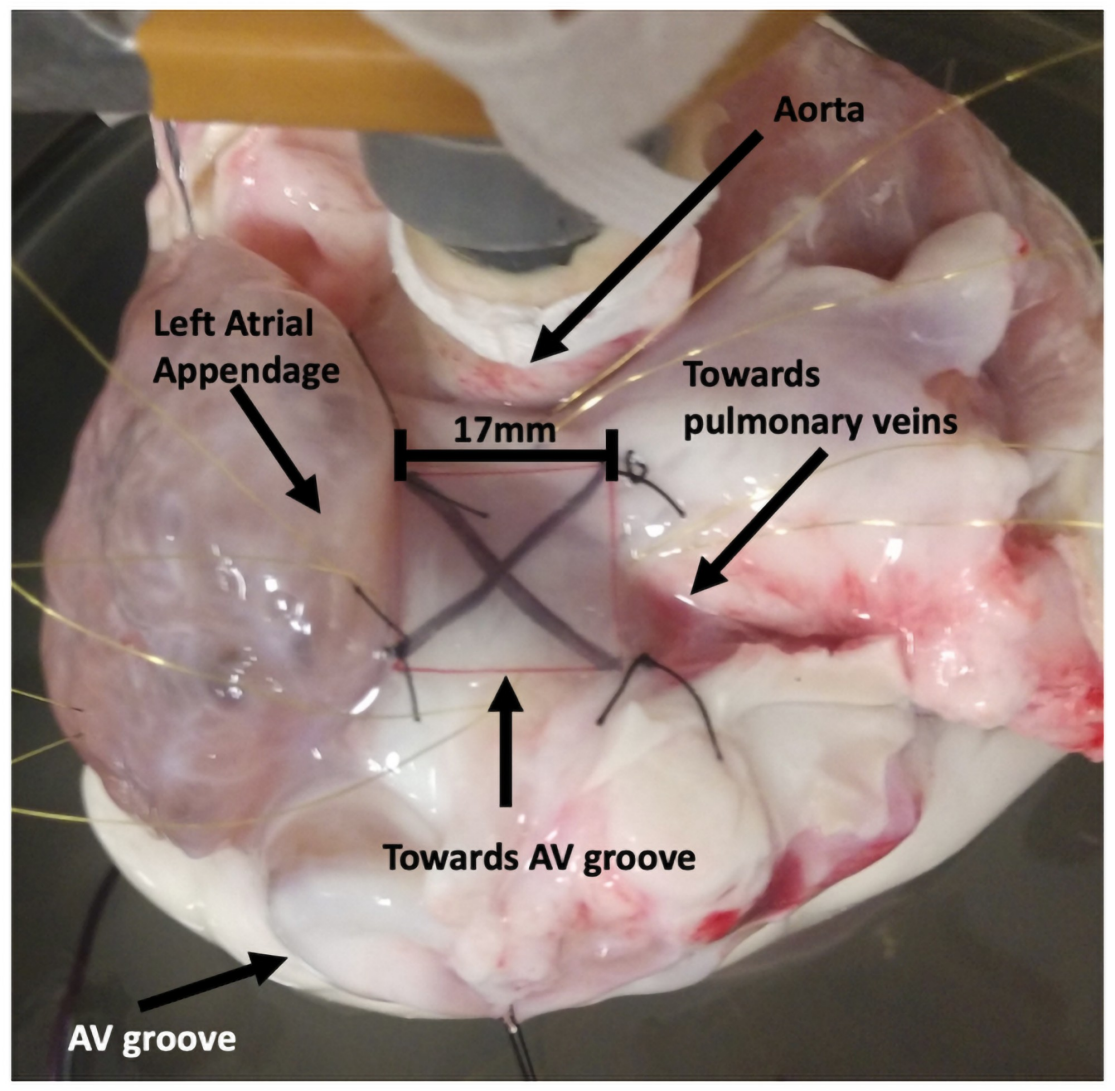
Preparation of the heart for the optical mapping. The X marks the mapped area. At the edges of the mapped region, the pacing wires are show at the 4 locations: top (Aorta), bottom (Towards AV groove), left (Left atrial appendage), and right (towards pulmonary veins).

In preparation for optical mapping, the heart was cleaned of excess tissue and rinsed with cold cardioplegic solution (NaCl 5.2mM, NaH_2_PO_4_-H_2_O 48*μ*M, MgCl_2_-6H_2_O 40*μ*m, KCl 160*μ*M, CaCl_2_-2H2O 72 *μ*M, NaHCO_3_ 0.96mM, Dextrose 11.20mM). The cleaned heart was transferred in a glass bowl, where is was perfused using a Langendorff setup with warm, oxygenated, and pH-balanced Tyrode solution. The perfused heart was positioned such that the optical mapping system (MiCAM05, Sci-Media, Costa Mesa, CA) with a lens (Plana 1.0x, Leica, 10450028, Germany) captured a square area of 17[*mm*] × 17[*mm*] of the left atrial roof. The boundaries of the captured area were used to guide the placement of pacing leads at the center of all four edges (Fig 1). The electromechanical uncoupler blebbistatin (8.55–17.1*μM*, ApexBio B1387) was added to the perfusionsolution to suppress the motion of the heart, and the voltage-sensitive fluorescent dye di-4-Anepps (25-75 *μg*, Sigma Aldrich D8064) was added to visualize the transmembrane potential.

Pacing was conducted from four sets of wires. Before pacing, any atrial arrhythmia was terminated by applying a monophasic shock to the atria. To highlight animals that maintained AF for 6 month but are kept in sinus during the study we use the term history of persistent AF. During the study, the rhythm was controlled by pacing. The pacing was delivered at 2 times the diastolic pacing threshold. The heart was paced from one wire set with an S1 train of 9 beats with a cycle length of 500-600ms, depending on the intrinsic rate, and a single S2. The minimal S2 was found by reducing the S2 delay in 10ms steps until loss of capture. If the S2 pacing resulted in AF, AF was terminated by a monophasic shock and the lowest S2 was found with a search starting with a non-captured short S2 and increasing the S2 delay in 10 ms steps.

The recording was managed with the software BV Workbench (Brainvision, Tokyo, Japan). Illumination was adjusted to reach 70% of saturation or the maximum stable light output of the light source (LEX2, Brainvison, Tokyo, Japan) at a recorded 3000 frames per second with 100 × 100 pixel.

### Image post processing and statistics

**Fig 2:**
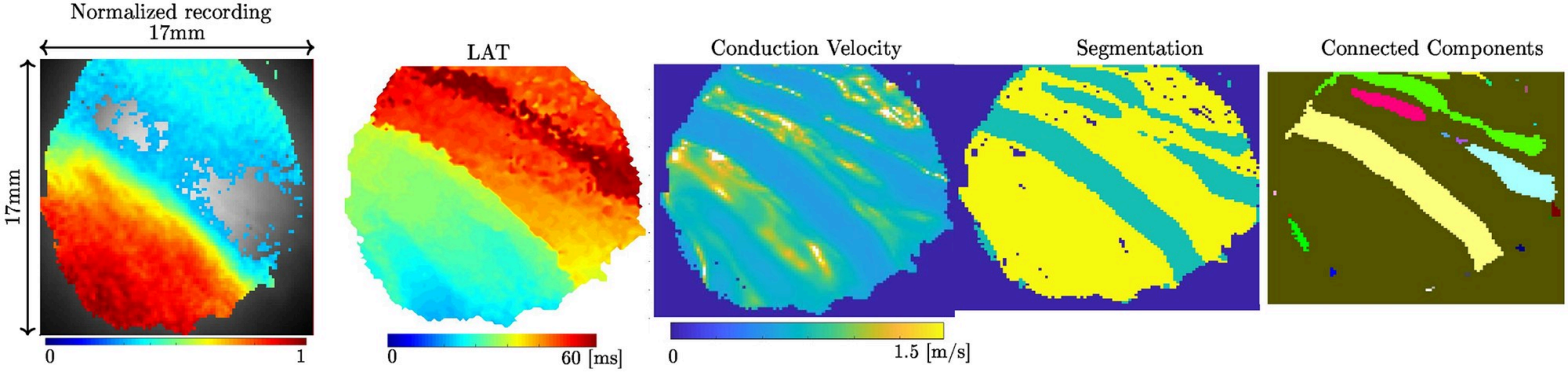
Processing of the recorded images. From left to right, **(A)** first the normalized and filtered recording, **(B)** then converted to local activation times, **(C)** the conduction velocity, **(D)** finishing with the thresholding of conduction velocity (less than 0.2 m/s). **(E)** Connected components marked in different colors.

The last recorded S1 and the following S2 are used for analysis. All visualization, processing, and calculation have been done with the open-source software Rhythm (Rhythm, v 1.2, Washington DC). To reduce noise, gaussian binning (kernel size 3 or 5) and bandpass (0-100Hz) filtering is used. For signal extraction, a drift correction and normalization were applied. The resulting image was masked to remove pixels with a low signal to noise ratio. From the masked images, local activation times and conduction velocities were calculated with Rhythm using the method described in [22]. Each pixel in the conduction velocity plot was classified as normal conduction or SC ($v<0.2\frac{m}{s}$), which results in a binary image. The image processing steps are shown in Fig 2. The first measurement is the percentage of all pixels in the region of interest (ROI) classified as SC, called percentage of SC. Then the binarized conduction velocity image is filtered for its connected components for SC (see Fig 2(E)) The connected component filter uses the direct neighboring horizontal and vertical pixel but not diagonal pixel. Each neighbor which exhibits SC conducting is counted to the current component. Components are grown until no further neighboring SC pixels are found. The process is repeated for every component. Components with more than one pixel in size are counted and their size is determined. Their size is given by the number of pixels in a particular component multiplied by 0.0289*mm*^2^ the area of a single pixel.

Statistical analysis was performed using the Stata-16.1 statistical software (StataCorp, College Station TX). Our data were a clustered sample dataset, with multiple observations per animal. We had four pacing lead positions used per animal, where 2 observations were collected (one S1 and one S2 pacing for the train variable) at each pacing lead position, so the total observations was n = 7 animals x 4 pacing lead positions x 2 train levels = 56—1 missing values = 55. We did not discard the animal with one unreliable observations, but instead used the remaining data for that animal. To account for data clustering, a multivariable mixed effects linear regression model was fitted, with animal as a random effect. Predictor variables were class (control vs persistent atrial fibrillation cases), train (S1 or S2 pacing), position of pacing lead (top, bottom, left, or right), train X class interaction, and position X class interaction, requiring 9 predictor terms in the model. For correct interpretation of main effects in the presence of interaction terms, post-fit marginal estimation Wald tests with mean centering were used to test the main effects, where adjusted means were compared while holding all other variables constant at their means. All comparisons were two-sided comparisons and considered significant if *p* <.05.

To avoid possible overfitting, where unreliable associations might be observed from having too many predictor variables for the sample size, we should have 10 independent observations per predictor variable. Our models used 9 predictor variables. In a clustered dataset, the number of independent observations is mimicked by the “effective sample size,” [23], which can be determined using the design effect [24], *D*_*eff*_ = 1 + [(average cluster size − 1) × *ICC*] where *ICC* is the intraclass correlation coefficient. The effective sample size = (#observations)/*D*_*eff*_ according to [25]. The average cluster size was *n* = 7.9 for all outcomes. The *ICC* was 0.420 for the “slow percentage” outcome, 0.150 for the “counts low condition areas” outcome, and 0.090 for the “mean area” outcome. Based on these, the effective sample sizes were *n* = 14 for “slow percentage”, *n* = 27 for “counts low condition areas” and *n* = 33 for “mean area.” Given these effective sample sizes, we recognize that there is a potential for overfitting in all of our models, especially in the “slow percentage” model.

## Results

The recorded activation for S2 pacing from the right for one animal was inconsistent with the expected activation. In that animal, the S2 pacing from the right did not originate from the pacing electrode. It appeared from the top first and the activation occurred on a shorter interval than the programmed S2. This leads to the assumption that the beat was not the programmed S2, but the spontaneous beginning of AF. Due to the uncertainty of the origin of this excitation, we excluded data from the right pacing position for this animal.

**Fig 3:**
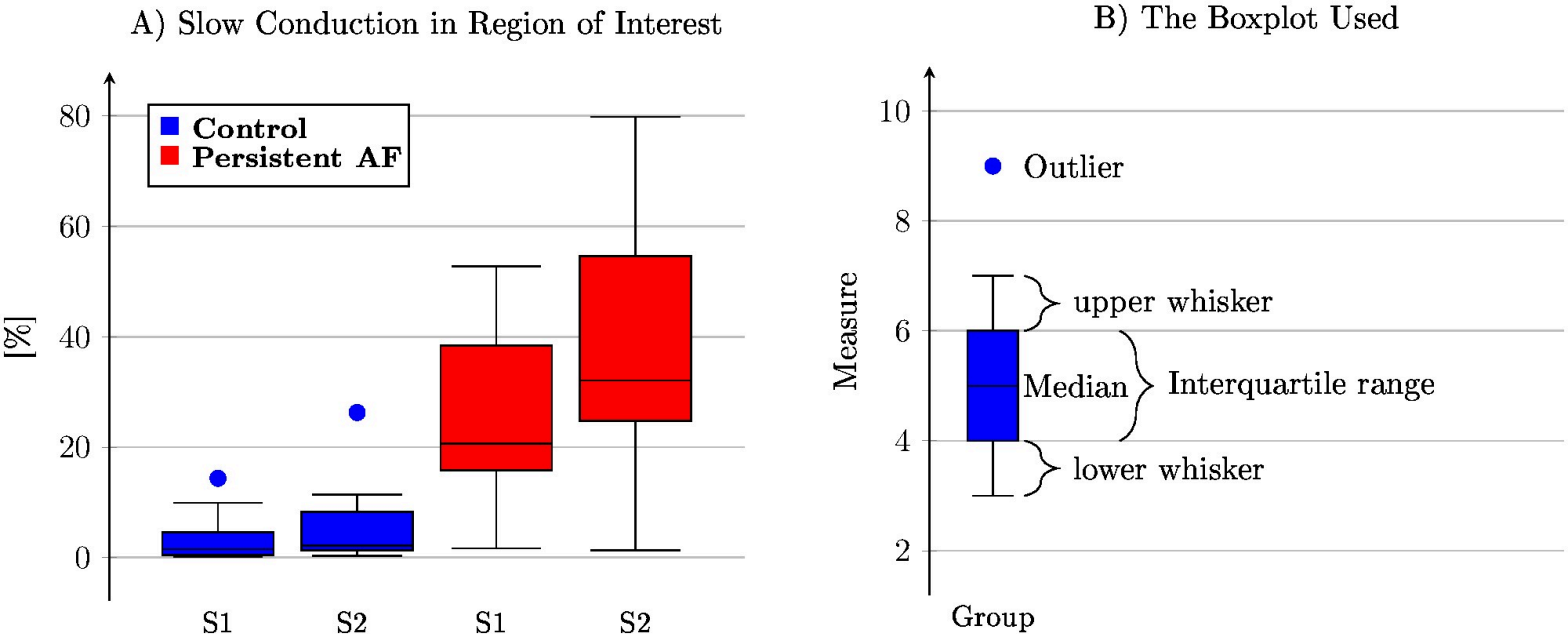
**(A)** The percentage of observed slow conduction in the region of interest. In control conditions, the premature excitation has a minor change, whereas for animals with a history of persistent atrial fibrillation, the change is significant. The expected increase in slow conduction regions between control and persistent AF animals is significant. **(B)** Description of the Boxplot used through the manuscript. The interquartile range (IQR) is from 25% to the 75% of all observed values. The upper whisker is defined as the largest observation which is smaller than *IQR* ⋅ 1.5+ (75 percentile of observed data). Similar, the lower whisker is the smallest observation that is larger than −*IQR* ⋅ 1.5+ (25 percentile of observed data). Observations that are not contained between the upper and lower whisker are considered an outlier. Boxplots are created with the LATEX package pgfplots. (http://pgfplots.sourceforge.net/).

In the first analysis of 55 activation recordings the SC area in the field of view is evaluated (see Fig 3). The linear mixed model demonstrates that in 24 (12 S1 beats and 12 premature S2 beats) control goat recordings, 4.4±4.6% of the total tissue in the field of view had SC. In 31 (16 S1 beats and 15 premature S2 beats) persistent AF goat recordings, the SC area increased significantly to a total of 30.6±4.0% of the field of view compared to control (*p* < 0.001). A less expected result is the correlation between the class and the train which is reflected in the fact at S1 and S2 pacing are not significantly different in control (an increase from 3.4±5.0% to 5.5±5.0%, *p* = 0.556) but becomes significantly different in persistent AF (an increase from 24.4±4.3% to 36.6±4.4%, *p* < 0.001). The shortest captured S2 beats are listed in Table 1.

**Table 1:** The shortest captured S2 in each animal and position, times are in [ms].

Animal	Top	Left	Bottom	Right	Mean±Std
Control 1	130	170	190	160	163±25
Control 2	160	180	170	260	192±46
Control 3	220	220	240	200	220±16
Mean±Std	170±46	190±26	200±36	207 + 50	
persistent AF 1	190	270	240	180	220±42
persistent AF 2	160	160	180	NA	167±12
persistent AF 3	170	180	190	250	198±31
persistent AF 4	210	210	240	200	287±17
Mean±Std	183±22	205±48	213±32	210±30	

**Fig 4:**
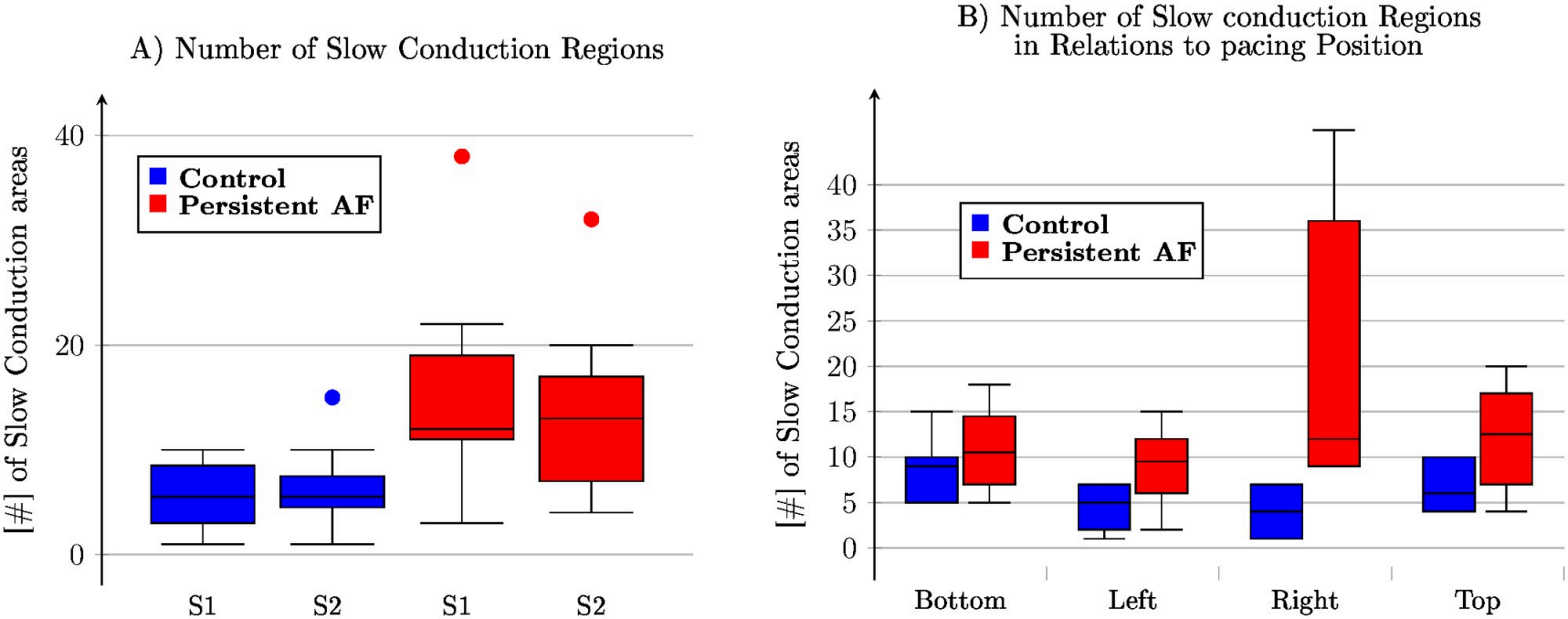
The number of slow conduction regions. **(A)** grouped by the type of paced beat and **(B)** by the position of the pacing electrode. The premature excitation does not change the number of slow conduction regions. Not all pacing positions result in the same number of slow conduction regions, however, the pacing position is not a significant factor.

Related to the percentage of SC is the number of observed not connected SC areas (Fig 4). In 24 control recordings, 6.0±1.8 SC areas were counted, while in the 31 persistent AF goat recordings, we observe a significant (*p* = 0.004) increase to 12.7±1.5 area s. While differences in the totals for different pacing positions (Fig 4) are observed (bottom 10.0±1.7, left 7.0±1.7, right 12.7±1.8, top 9.7±1.7) all four positions are statistically the same. Similarly, there is no significant change between S1 and S2 pacing for control (5.7±2.1 vs. 6.4±2.1, *p* = 0.769) and persistent AF (11.6±1.8 vs. 13±1.9, *p* = 0.242).

**Fig 5:**
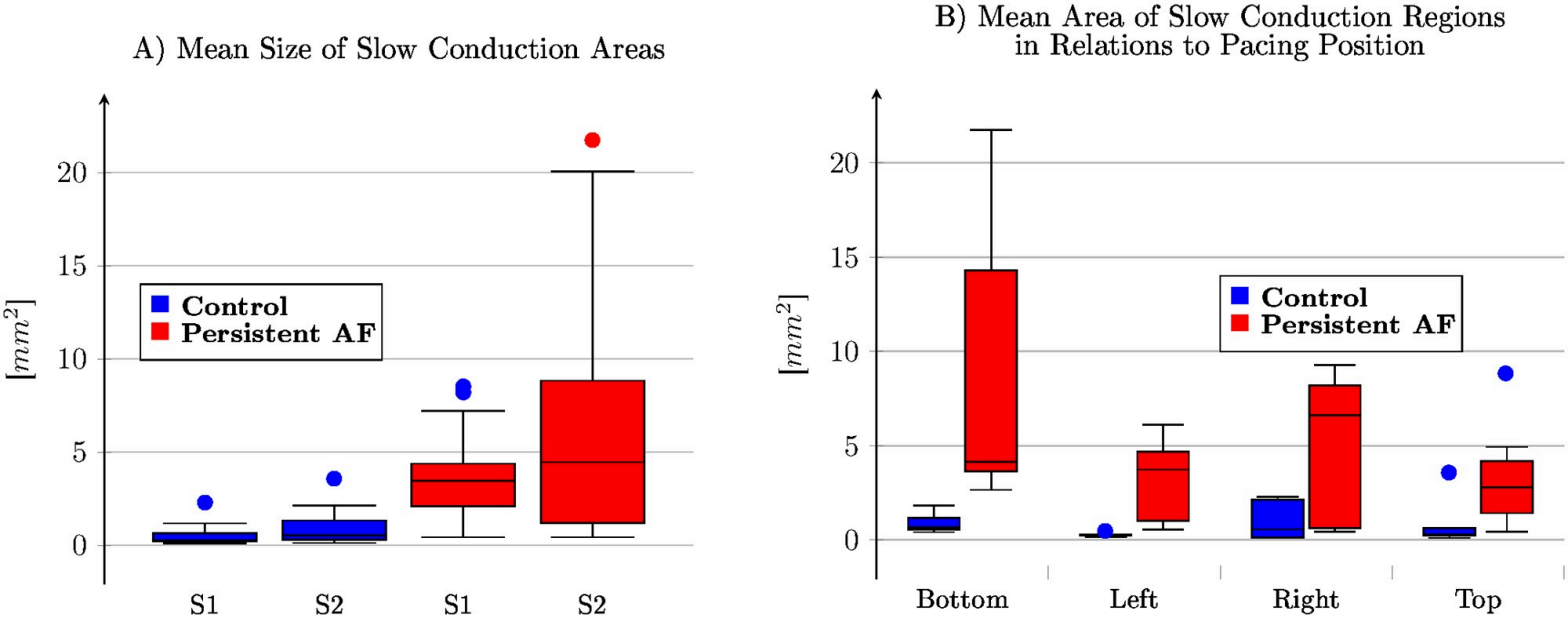
Means size of slow conduction area. **(A)** A significant difference between control and persistent atrial fibrillation animals was detected. However, within each class, premature beats do not significantly change the size.**(B)** Effect of the pacing position on the mean area size. The bottom and right positions appear similar in persistent AF animals, as do the left and top positions.

Comparing the average size of SC areas for persistent AF animals shows a significant change (*p* = 0.014) and the absolute change is more noticeable from 3.70±0.89[*mm*^2^] to 6.36±0.91[*mm*^2^]. Within control animals the average size of SC areas increases from 0.53±1.03[*mm*^2^] to 0.95±1.03[*mm*^2^] for premature excitation which is not significant (*p* = 0.735). Comparing the 24 control samples 0.74±0.82 [*mm*^2^] and 30 persistent AF samples 5.01±0.72 [*mm*^2^] shows a significant (*p* < 0.001) difference (Fig 5). In contrast to the number of SC regions, the mean size of SC regions depends on the pacing position (Fig 5). While the position bottom and right (5.19±0.88 [*mm*^2^] vs. 3.28±0.91 [*mm*^2^], *p* = 0.101) show larger SC areas, the position top and left (2.2±0.88 [*mm*^2^] vs. 1.90±0.88 [*mm*^2^], *p* = 0.800) show small SC areas. The statistical test shows that the four position are not from the same distribution (*p* = 0.016).

**Fig 6:**
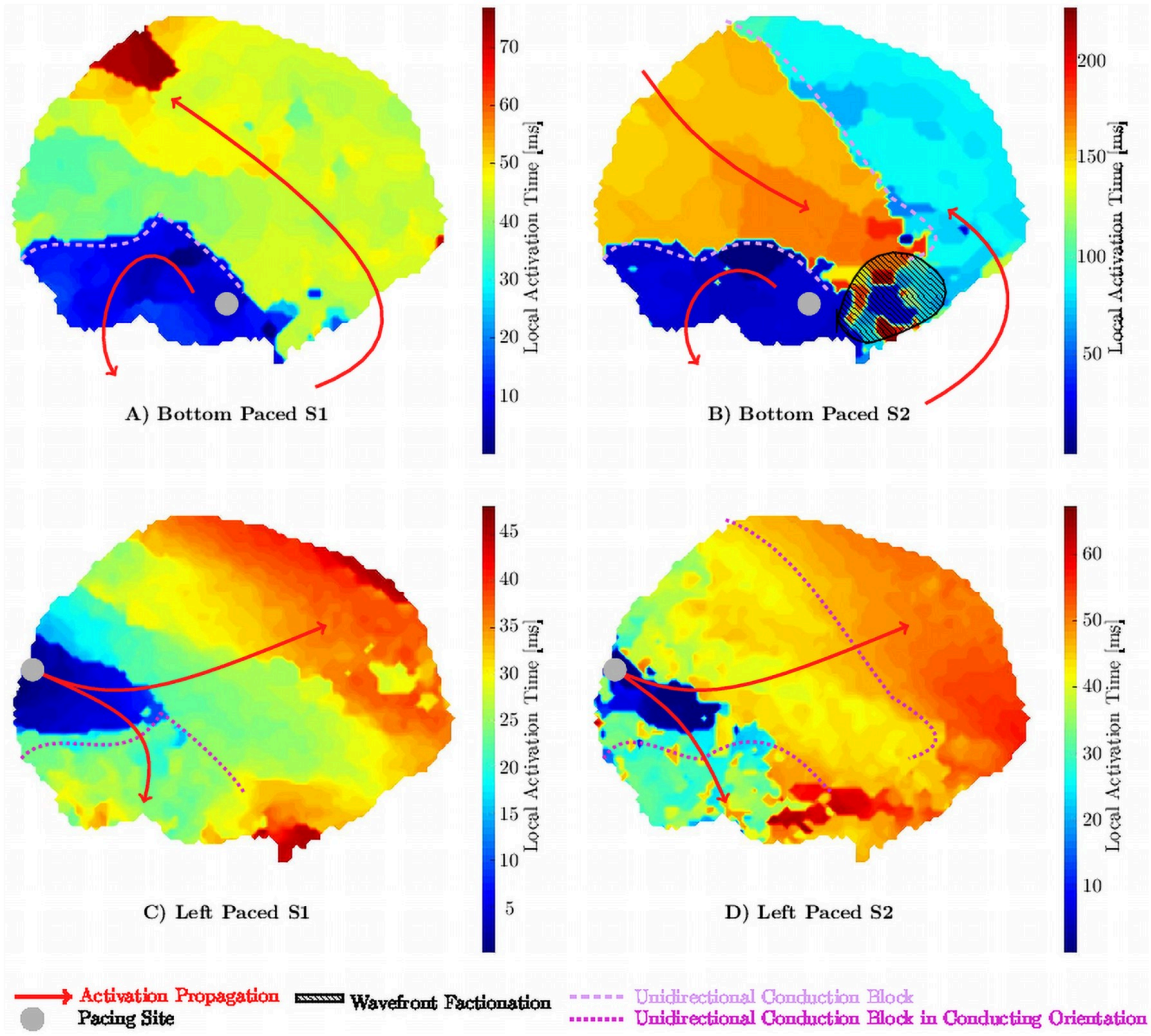
Local activation times showing unidirectional conduction block. A line of blockage in the lower half is observed (dashed line) when pacing from the bottom **(A)** and **(B)**. In response to a premature beat **(B)** an additional line of blockage develops spanning the field of view at about 45 degrees. In comparison, pacing from the left in physiological **(C)** or premature **(D)** condition does not show a significant blockage.

Dependency on the direction of propagation is demonstrated in Fig 6. Two perpendicular pacing points bottom and left are shown for S1 and S2 beats. The pacing from the bottom shows lines of conduction block in S1 and S2, however, for the S2 paced beat a new line of conduction block develops spanning almost the ROI. This newly developed line of conduction block changes the activation pattern dramatically. Additionally, a small region of wavefront fractionation developed. The lower panels in Fig 6 show the same experiment but with pacing from the left. Parts of the conduction block observed in bottom pacing (Fig 6A) disappear and propagate freely. No new line of blockage develops in response to the premature beat.

## Discussion

In this manuscript, we investigate if new slow conduction areas are created in response to a premature excitation in atrial tissue. From optical activation mapping of ex-vivo Langendorff perfused control and persistent AF in goat hearts, we established the overall percentage of SC, the number of connected components, and their average size. This data shows an increase of overall slow conduction driven by enlarging existing SC areas and not by creating new areas of SC.

The clinically used premature pacing of an S1-S2 train does exacerbate SC areas, confirming the effectiveness of this approach. Unexpectedly, the percentage of SC in S1 and S2 does not change significantly for control animals. Contributing factors might include the higher overall conduction velocity in control animals, which is well characterized in the literature [8, 26] and observed in our study (data not shown). It is also likely that healthy tissue has larger functional reserves to compensate for premature excitation. As a consequence, premature pacing might be less efficient exacerbating SC areas in paroxysmal AF. Conversely, tissue with a history of persistent AF exhibits a significant increase in SC following premature excitation.

Considering that premature excitation in controls does not lead to a significant change in overall SC, it is not surprising that neither the count of SC areas nor the average size of SC areas changed in response to premature excitation. This reinforces the idea of larger functional reserves in control animals.

In contrast, in persistent AF animals, the percentage of SC increased significantly. The increase cannot be explained by the number of connected SC components, as they remained similar. However, the average size of a slow conduction region increased from 3.7 [*mm*^2^] to 6.36 [*mm*^2^], almost doubling in area. This finding supports the hypothesis that SC regions are fixed in location, but recruit additional surrounding tissue in response to a premature excitation. This could indicate that areas of tissue remodeling, of structural or ionic nature, have a core region that gradually expands towards the surrounding tissue. In longer pacing cycle length (S1 beats), the distal sections are still able to compensate for the tissue remodeling, but in shorter S2 excitation this is no longer possible.

We demonstrated that premature excitation is important and, importantly, that attention should also be given to the direction of propagation. We found that experiments with persistent AF animals were sensitive to the direction of activation propagation; while one direction propagates seemingly undisturbed, another can show large areas of SC (an example is shown in Fig 6. Several factors contribute to this phenomenon. First, the predominant myocardial fiber orientation is likely to impact this measurement. Propagation transverse to fiber orientation is typically slower than propagation in the same direction as fiber orientation. Thus, with a smaller perturbation, it can drop below the SC threshold. A further contribution comes from a unidirectional conduction block. Conduction block and unidirectional conduction block can develop as a consequence of sudden changes in the fiber orientation, as often observed in the atria or in tissue with high fibrosis. Overall, this suggests that in order to identify SC areas, it is important to test multiple excitation sites in addition to finding the shortest S2 possible. This suggestion confirms a previous suggestion from a case report of Starreveld and de Groot who concluded that pacing from different locations and pacing cycle lengths may help in identification non-anisotropic tissue and thus substrate identification for AF [18].

Our results agree with other findings suggesting that persistent AF increases fractionating and slowing the spread of activation. Throughout all our analysis, the persistent AF group showed more SC compared to the control group, which has been attributed to different causes. Frequently, slow conduction does coincides with fibrotic areas [8]. Other hypotheses include disarrangement of myocardial fiber orientation which could lead to fractionation [27] and a sudden change in myocardial wall thickness which has been linked to rotor attraction in computational models [28].

In conclusion, this work demonstrated that premature excitation does not generate new SC areas, but expands already existing areas in hearts with a history of persistent atrial fibrillation. Limitations of the study include that only a small area of tissue has been studied. There is also a possibility that our results could be affected by overfitting. The potential for this is especially high in our “slow percentage” model results, which had the smallest effective sample size. However, the results are consistent with the anticipated effect, which increases confidence that the conclusions presented are correct. Further limitations include that the fiber orientation for the area is unknown. Evaluating fiber orientation would provide information as to whether the conduction block occurs parallel or perpendicular to the fiber direction.
